# Serotonin Reuptake Inhibitors in Obstructive Sleep Apnea: Associations in People with and without Epilepsy

**DOI:** 10.1155/2018/7247605

**Published:** 2018-08-28

**Authors:** Jocelyn Y. Cheng

**Affiliations:** NYU Comprehensive Epilepsy Center, NYU Comprehensive Epilepsy Center-Sleep Center, Department of Neurology, NYU School of Medicine, 223 E. 34th Street, New York, NY 10016, USA

## Abstract

**Purpose:**

Positive airway pressure remains the gold-standard treatment for OSA, but many are intolerant. The neurotransmitter serotonin is involved in respiratory control. Evidence exists for SRIs in reducing OSA severity in the general population and ictal hypoxemia and seizure-induced respiratory arrest in people with epilepsy (PWE). However, the association between SRIs and OSA severity has not been studied in populations consisting of both groups. This study aims to determine if SRIs are associated with OSA severity in both PWE and people without epilepsy (PWO) and whether differences exist between the two groups.

**Methods:**

A retrospective study of adults with OSA was conducted. Subjects were categorized as PWE or PWO and for the use (+SRI) or absence (-SRI) of an SRI. The primary outcome was OSA severity relative to SRI status. OSA severity as a function of SRI status was also compared between PWE and PWO and within the PWE and PWO cohorts. Oxygen saturation nadir was a secondary outcome measure. Statistical adjustment of pertinent characteristics was performed.

**Results:**

There were 125 subjects (57 PWE, 68 PWO, 80 –SRI, and 45 +SRI). +SRI was associated with reduced odds of severe compared to moderate OSA, in unadjusted and adjusted analysis. Compared to PWO, PWE demonstrated a more robust association between OSA severity and +SRI. When analyzed as separate cohorts, only PWE demonstrated reduced OSA severity, with adjustment for age (OR:0.140, CI:0.021-1.116, and p=0.042). Oxygen saturation nadir was not significant in any model.

**Conclusions:**

SRIs represent a potential treatment option for OSA and may demonstrate a more robust association with reduced OSA severity in PWE compared to PWO.

## 1. Introduction

Obstructive sleep apnea (OSA), characterized by the reduction of airflow during sleep, is a common disease, affecting approximately 2-7% of adults in the general population [1]. Prevalence rates in specific groups are even higher, ranging from 7-63% in those with depression to 13-80% in people with epilepsy (PWE) [2, 3]. Untreated OSA is associated with elevated mortality and morbidity, including hypertension, stroke, and metabolic dysfunction [1]. In PWE, additional risks include seizure exacerbation and increased epileptogenicity [3, 4]. While the gold-standard for treatment of OSA generally remains devices administering positive airway pressure (PAP) [5, 6], many patients are unable to tolerate this [7], and alternative therapies have been investigated. One potential therapy is the use of serotonin reuptake inhibitors (SRIs).

Serotonin is involved in the control of respiration at multiple sites in the central (CNS) and peripheral nervous system (PNS) [8, 9]. Several studies have investigated the role of serotonin in OSA, with SRIs demonstrating potential efficacy at reducing OSA severity in animal models and the general population [10–12]. Increasing evidence also suggests that SRIs may impact breathing-related problems associated with seizures by reducing ictal hypoxemia in medically refractory PWE and seizure-induced respiratory arrest in the DBA/1 mouse model of sudden unexpected death in epilepsy (SUDEP) [13, 14].

As OSA is a prevalent condition associated with significant mortality and morbidity, in which a substantial proportion of individuals remain untreated due to intolerance to PAP, investigating alternate therapies would benefit not only individuals with OSA but also the population at large, in terms of healthcare-related costs on potentially preventable medical consequences associated with untreated OSA [15, 16]. For PWE, the benefits of treatment with a SRI may be even broader, as PWE suffer from seizure-related respiratory phenomenon in addition to possessing a higher prevalence of OSA, each of which may be potentially ameliorated by SRIs.

However, while some evidence exists for the utility of SRIs in OSA in the general population, this has not been specifically studied in PWE. Thus, the objectives of this study are to determine (1) whether the use of SRIs is associated with OSA severity in a population consisting of both PWO and PWE and (2) whether OSA severity, as a function of SRI status, differs in PWE compared to PWO.

## 2. Material and Methods

This study was approved by the institution IRB. Subject consent was waived due to the retrospective nature of the research, as, for this type of study, formal consent is not required. The study was performed in accordance with the ethical standards as laid down in the 1964 Declaration of Helsinki and its later amendments or comparable ethical standards.

A retrospective study of people with (PWE) and without (PWO) epilepsy was conducted. Inclusion criteria consisted of adults ≥ 18 years of age with depression who underwent diagnostic polysomnography for suspected sleep apnea at an academic sleep center between January 1, 2011, and January 1, 2016. Subjects with depression were included in order to increase the likelihood of concomitant SRI prescription for the purposes of this study. Potential subjects were identified via electronic search for the ICD-10/ICD-9 and CPT codes for sleep apnea, PSG, depression, and epilepsy. Search terms for sleep apnea included the ICD-10 code G47.3, and the ICD-9 codes for obstructive sleep apnea (327.23), sleep related nonobstructive alveolar hypoventilation (327.27), obesity hypoventilation syndrome (278.03), sleep related hypoventilation/hypoxemia (327.26), primary central sleep apnea (327.21), Cheyne Stokes breathing pattern (786.04), central sleep apnea/complex sleep apnea (327.27), other sleep apnea (327.29), apnea not elsewhere specified (786.03), and unspecified sleep apnea (327.20). Although only subjects with OSA were selected for the study, broader search terms encompassing any form of sleep apnea were utilized in order to capture potential subjects who may have been miscoded. Polysomnography search terms included ICD-10-PCS 4A1ZXQZ, ICD-9-CM 89.17, and CPT code 95807 (sleep study, simultaneous recording of ventilation, respiratory effort, ECG or heart rate, and oxygen saturation, attended by a technologist). Depression search terms included the ICD-10 codes F32 and F33, ICD-9 codes 311, 296.2, and 296.3, and epilepsy codes consisting of ICD-10 G40 and ICD-9-CM 345.

Charts were subsequently reviewed to confirm whether diagnostic PSG had been completed and whether a depression code was present, during the designated dates. Extended EEG was not performed with PSG. Charts were individually reviewed to verify the diagnoses of epilepsy (based on the 2014 International League Against Epilepsy operational clinical definition of epilepsy [17]) and OSA (with PSG scoring based on the Center for Medicare and Medicaid Services criteria [18]) and for the use and identity of SRIs, at the time of baseline PSG.

All subjects were divided into two groups by exposure: those taking (+SRI) and those not taking (-SRI) an SRI during PSG. Within these exposure groups, subjects were subdivided into PWE and PWO. Baseline characteristics were collected for age at the time of diagnostic PSG, gender, body mass index (BMI), hypertension (HTN), diabetes (DM), congestive heart failure (CHF), chronic obstructive pulmonary disease/asthma (COPD), epilepsy diagnosis, epilepsy syndrome (focal, generalized, or undetermined), tobacco use, alcohol abuse, illicit substance abuse, and a record of opioid, benzodiazepine, nonbenzodiazepine, and antiepileptic drug (AED) use at the time of baseline PSG.

The AHI was stratified into three categories of severity: mild OSA (5<AHI<15), moderate OSA (15≤AHI<30), and severe OSA (AHI≥30). Based on data from the Wisconsin Sleep Cohort study, which demonstrated that a significant risk of hypertension occurs at AHI values of approximately 30 or greater [19] and subsequent recommendations from the American Academy of Sleep Medicine Task Force [20], severity levels of OSA, rather than continuous AHI values, were analyzed. The primary outcome measure was severe OSA, with O2 nadir (O2≤88%) as a secondary outcome.

Four distinct analyses were conducted from the study sample. The relationship between OSA severity and SRIs was assessed for (1) the entire study sample (consisting of PWO and PWE), (2) PWO compared to PWE, (3) PWO only, and (4) PWE only.

### 2.1. Statistical Analysis

Baseline characteristics were analyzed using Pearson's *χ*^2^ for categorical variables and independent samples t-testing, two-tailed, equal variances not assumed, for continuous variables. Binominal logistic regression assessed whether significant associations exist between OSA severity and SRIs, with OSA severity as the dependent variable and SRI as the independent variable. Adjustment was performed in logistic regression models for significantly different baseline covariates only, in order to avoid overfitting. A p<0.05 was considered statistically significant.

## 3. Results

### 3.1. Study Population

After screening, the first ninety PWE who potentially met inclusion criteria were selected and compared via convenience sampling to the first 90 PWO screened who also potentially met inclusion criteria, for a potential study population of 180 subjects. Of these individuals, 55 were not eligible (diagnostic PSG was not done or did not meet PSG scoring criteria for OSA), resulting in a total study sample of 125 subjects: 57 PWE and 68 PWO.

Across the entire cohort (PWE and PWO), 45 were +SRI and 80 were –SRI. Baseline characteristics comparing +SRI and –SRI exposure groups are listed in Table 1. The +SRI group was older and more likely to use AEDs than –SRI. Among PWE and PWO, PWO were older, more likely to have DM and COPD/asthma and less likely to use AEDs than PWE (Table 2).

### 3.2. Outcomes

Four separate analyses were conducted, each examining the relationship between OSA severity and SRIs in different populations within the study sample: (1) PWO and PWE, (2) PWO compared to PWE, (3) PWO only, and (4) PWE. The mean AHI of each OSA severity category is listed by subpopulation (SRI and epilepsy status) in Table 3.

### 3.3. Total Cohort: PWE and PWO (Table 4)

Unadjusted and adjusted analysis, controlling for the significant covariates of age and AEDs, was performed.

+SRI was less likely to have severe OSA than -SRI compared to moderate OSA in univariate and multivariate analysis. There was no association with SRI exposure in comparing the outcome of severe versus mild OSA.

Oxygen saturation nadir was not associated with SRI exposure, in unadjusted and adjusted analysis (data not shown).

### 3.4. PWE versus PWO (Table 5(a))

Subgroup analysis was performed to determine whether PWE and PWO demonstrated similar or different associations with SRI exposure to outcome. Univariate and multivariate analysis adjusted for covariates which were significantly different at baseline between PWE and PWO: age, DM, COPD/asthma, and AED use.

The +SRI group with epilepsy was less likely to have severe OSA compared to moderate OSA, after multivariate adjustment. Severe compared to moderate OSA was also less likely when AED use was accounted for.

The outcomes of severe compared to mild OSA and O2 nadir (data not shown) again did not demonstrate any associations.

### 3.5. PWO (Table 5(b))

At baseline, +SRI was more likely to have COPD/asthma (*χ*^2^ 4.032, p=0.045), which was controlled for in logistic regression analysis.

For PWO, there was no association between the outcome measures of OSA severity and O2 nadir (data not shown) and SRI exposure, without and with adjustment.

### 3.6. PWE (Table 6)

At baseline, +SRI was more likely to be older (60.70±8.64 versus 51.09±13.55, p=0.002, CI -15.50- -3.71), which was controlled for in logistic regression analysis.

For PWE, +SRI was less likely to have severe compared to moderate OSA after adjustment for age. There was no association between SRI exposure and O2 nadir (data not shown).

## 4. Discussion

The main findings of this study are that, in people with depression, (1) SRI use is associated with reduced OSA severity and, (2) in PWE but not PWO, SRI use is associated with reduced OSA severity. This suggests that the association between SRIs and measures of OSA severity is more robust in PWE compared to PWO, as this outcome difference was also present when directly comparing PWE to PWO who used SRIs.

The first finding is in line with a large body of evidence, in which several human trials have shown that SRIs ameliorate measures of OSA. Treatment of OSA was compared in a randomized, crossover trial using either fluoxetine or protriptyline for 4 weeks. Baseline AHI decreased nearly 40% with both drugs, driven by improvement of the non-REM (NREM) AHI. There was no change in oxygen saturation. However, individual outcomes were widely variable, with only half of patients demonstrating a good response and not necessarily to both drugs [21]. Prasad et al. studied the effect of fluoxetine, ondansetron, or both, versus placebo in patients with OSA over 28 days of treatment. Overall AHI decreased by a mean of 12.9, or approximately 40.5%, from baseline at day 28 for patients on both fluoxetine and ondansetron, but not for either drug alone. This reduction in AHI remained significant for both NREM and REM sleep, and there was no improvement in oxygen saturation [12].

In a double-blind, randomized, placebo-controlled crossover study, response to paroxetine administered for 6 weeks was studied in 20 men with OSA. Paroxetine reduced the NREM apnea index by 35%, but no significant effect was found for the hypopnea index or during REM sleep. Oxygenation was not reported [22]. Assessing the utility of paroxetine from a different perspective, Berry's group demonstrated that acute administration of paroxetine 4 hours prior to polysomnography in 8 men with severe OSA increased genioglossus peak muscle activity without significant effect on AHI or oxygen saturation [23].

Mirtazapine, a mixed 5-HT2/5-HT3 and alpha-2A antagonist, initially demonstrated promising results in a randomized, double-blind, placebo-controlled, 3-way crossover study of 12 adults with OSA, where patients were administered placebo, mirtazapine 4.5 mg, or mirtazapine 15 mg daily for 7 days. Mirtazapine at either dose significantly reduced NREM and REM AHI, as well as oxygen desaturation index [24]. However, in two larger trials of 60 and 65 OSA patients randomized to higher doses of mirtazapine for 2 and 4 weeks, respectively, no measures of sleep apnea improved, and weight gain was significantly greater for those on mirtazapine in both trials [25].

It is well-established that serotonin plays an integral role in respiratory control [8, 9]. In the central nervous system (CNS), serotonin has been demonstrated to stimulate 5-HT2A and/or 5-HT2C upper airway dilator motor neurons and to activate clusters of respiratory neurons in the brainstem mainly via the 5-HT1A, 5-HT2A, or 5-HT2C receptors [7]. Complementarily, simulation of apnea in rats lowers genioglossus tone and increases diaphragmatic activity [26]. Serotonergic neurons also promote arousal [27], which may be critical in responding to hypercapnia.

Peripherally, however, serotonin appears to play a large inhibitory role in the control of respiration, where inhibitory effects at 5-HT2A and/or 5-HT2C and 5-HT3 dominate at the nodose ganglion [9]. Peripherally acting 5-HT3 agonists have been shown to exacerbate spontaneous central sleep apneas during stage REM [28]. Conversely, administration of ondansetron, a 5-HT3 antagonist, reduced central sleep apneas in animal models via peripheral effects at the nodose ganglion [29, 30].

Differential effects are also influenced by duration of exposure. In rat models, SRI administration for 14 or 23 days was associated with higher serotonin levels [31] and differing respiratory rate alterations [32] compared to acute administration. Posited mechanisms for the differences in acute versus chronic administration of SRIs include time required to achieve steady state concentrations in the CNS [33], desensitization of somatodendritic autoreceptors [34], and regulation of BDNF gene expression [35].

The selectivity of each SRI for 5-HT versus norepinephrine reuptake may also result in variability. In the present study, subjects were exposed to four SRIs: escitalopram (N=15), fluoxetine (N=13), sertraline (N=12), and paroxetine (N=2). Of these, 5-HT selectivity is the greatest with escitalopram, followed by sertraline, paroxetine, and fluoxetine [36, 37]. Complicating this, the association between OSA and sertraline or escitalopram has not been well studied.

Thus, serotonergic effects on respiration are complex and likely depend on multiple factors, including the degree of activity and selectivity at different receptor subtypes, anatomical location of response, and duration of exposure. While the literature generally suggests that SRIs have a positive modulating effect on OSA, the interaction between these variables likely accounts for the somewhat mixed results that SRIs have demonstrated in human studies of OSA.

Overall, human trials suggest that SRIs ameliorate measures of OSA, in accordance with the results of this study. Of interest, this association was present when those with moderate OSA were compared to the severe groups, but not when the mild severity group was compared to those with severe OSA. This suggests that SRIs may only modulate OSA after it reaches a certain degree of severity.

The other main finding of this study was that the association between SRIs and OSA severity is stronger in PWE compared to PWO. This is notable in that, to the author's knowledge, no data exists evaluating the effect of SRIs on OSA in PWE, with the exception of one small case series in which protriptyline, a norepinephrine and serotonin reuptake inhibitor, was used [38]. However, the objective of the report was to describe the effect of OSA treatment on seizure frequency, and results regarding OSA outcome were only incidental. Of 7 patients in the series, 4 were on CPAP, 1 had a tracheostomy, 1 was treated with protriptyline, and 1 was treated with protriptyline followed by CPAP. Neither of the patients on protriptyline demonstrated a significant change in AHI.

The stronger association between OSA severity and SRIs in PWE is not a given, as there is evidence that PWE demonstrate serotonergic deficiency at baseline. In PWE, PET studies have shown decreased 5-HT1A receptor binding [39] and low concentrations of CSF 5-hydroxyindoleactic acid, a 5-HT metabolite [40]. Animal data has also supported this [41]. Thus in PWE, dysfunctional serotonergic levels may reduce the ability of SRIs to impact OSA, if at all.

In this present study, PWE demonstrated a stronger association between OSA severity and SRIs compared to PWO, both in direct comparison and when analyzed separately as a group. In support of this, when examined as separate cohorts, PWO did not demonstrate a significant effect between SRI use and OSA severity, regardless of adjustment, whereas PWE did, after univariate adjustment for age. The reason(s) for this difference is unclear. However, one potential mechanism may be that, due to lower serotonergic function and/or concentration, PWE possess a lower threshold for serotonergic effects compared to PWO.

Significant baseline covariates which were identified and adjusted for in this study included those commonly associated with OSA in larger cohorts, including diabetes, older age, and pulmonary disease [42, 43]. In addition, AEDs, which are not routinely studied in OSA series, attenuated the association between SRIs and OSA severity in the combined PWE and PWO. This is congruent with the more limited literature available regarding AEDs and OSA, which have typically been examined in PWE. For example, Foldvary-Schaefer's group found that a higher AED standardized dose was predictive of OSA in multivariate models in PWE [44]. Of note, AEDs are not only used for epilepsy; primary indications for certain AEDs include migraine headache and neuropathic pain, which may be why PWO were also prescribed AEDs.

There are several limitations to this research. Although diagnostic accuracy was controlled for by confirmation of OSA and epilepsy diagnoses via individual chart review and significantly different baseline comorbidities were adjusted for, the limitations of retrospective research remain. ICD 10/9 and CPT codes for diagnoses other than OSA and epilepsy were not verified and may have been inaccurate. Adjustment for multiple potential confounders was not performed due to small sample size and incomplete data. This included seizure frequency, type(s) of AEDs used, SRI duration of use, SRI dose, type of SRI used, and medication adherence. Only associations could be established. As a single site study with a limited number of subjects, all of whom suffered from depression, the results may not be generalizable, and sampling error may have skewed results. Nonetheless, the findings of this study merit further investigation. As evidence exists for the role of serotonin in seizure control, additional benefits may also apply in PWE [45, 46]. Future studies might include basic research examining the effect of targeting particular subtype(s) of serotonin receptors, prospective, randomized, placebo-controlled trials in larger populations, and trials focusing on PWE and the impact that SRIs may have on seizure control as well as OSA.

## 5. Conclusions

In summary, reduced OSA severity is associated with SRI use in populations consisting of people with and without epilepsy. For individuals with epilepsy, the relationship between reduced OSA severity and SRI use may be even more robust. SRIs, a drug class already well-established for use in conditions such as depression, may represent a potential and readily available treatment option for OSA in people with and without epilepsy and warrant further investigation.

## Figures and Tables

**Table 1:** Baseline characteristics.

	-SRI (N=80) N (%)	+SRI (N=45) N (%)	*χ* ^2^	p
Gender (male)	44 (55)	26 (58)	0.090	0.764
Hypertension	21 (26)	18 (40)	2.537	0.111
Diabetes	15 (18.75)	11 (24)	0.567	0.451
Congestive Heart Failure	7 (8.75)	3 (6.67)	0.170	0.680
Stroke/TIA	15 (18.75)	8 (17.78)	0.018	0.893
COPD/asthma	13 (16.25)	13 (28.9)	2.793	0.095
Epilepsy	34 (42.5)	23 (51)	0.861	0.353
Focal epilepsy	31 (38.75)	18 (40)	0.019	0.891
Generalized epilepsy	2 (2.5)	2 (4.44)	0.352	0.553
Undetermined epilepsy	1 (1.25)	3 (6.67)	2.728	0.099
Tobacco	4 (5)	0 (0)	2.324	0.127
Alcohol	0 (0)	0 (0)	------	------
Drug Abuse	3 (3.75)	1 (2)	0.217	0.641
Opioids	3 (3.75)	1 (2)	0.217	0.641
Benzodiazepines	17 (21.25)	13 (28.9)	0.921	0.337
Non-benzodiazepines	9 (11.25)	4 (9)	0.172	0.678
AED*∗*	41 (51.25)	32 (71.11)	7.898	0.048

	Mean ± SD	Mean ± SD	CI	P

Age (years)*∗*	57.73±14.43	62.51±10.36	-9.21- -0.366	0.034
Body Mass Index	30.86±6.13	30.95±5.91	-2.30-2.14	0.942

SRI: serotonin reuptake inhibitor, TIA: transient ischemic attack, COPD: chronic obstructive pulmonary disease, and AED: antiepileptic drug; *∗*: p<0.05.

**Table 2:** PWO and PWE: significantly different baseline characteristics.

	PWO (N=68) N (%)	PWE (N=57) N (%)	*χ*2	p
Gender (male)	37 (54)	33 (58)	0.153	0.696
Hypertension	26 (38)	13 (23)	3.438	0.064
Diabetes*∗*	19 (28)	7 (12)	4.616	0.032
Congestive Heart Failure	8 (12)	2 (3.51)	2.872	0.090
Stroke/TIA	15 (22)	8 (14)	1.330	0.249
COPD/asthma*∗*	20 (29)	6 (10.5)	6.713	0.010
Tobacco	4 (5.88)	0 (0)	3.464	0.063
Alcohol	0 (0)	0 (0)	------	------
Drug Abuse	2 (2.94)	2 (3.51)	0.032	0.857
Opioids	4 (5.88)	0 (0)	3.464	0.063
Benzodiazepines	18 (26.47)	12 (21)	0.499	0.480
Non-benzodiazepines	9 (13.24)	4 (7.02)	1.286	0.257
AED*∗*	18 (26.47)	55 (96.49)	65.712	0.001

	Mean ± SD	Mean ± SD	CI	P

Age (years)*∗*	63.21±12.67	54.97±12.65	3.74-12.74	0.001
Body Mass Index	31.54±6.48	30.12±5.39	-0.68-3.52	0.301

SRI: serotonin reuptake inhibitor, TIA: transient ischemic attack, COPD: chronic obstructive pulmonary disease, and AED: antiepileptic drug; *∗*: p<0.05.

**Table 3:** Mean AHIs of OSA severity by SRI and epilepsy status.

OSA Severity		AHI: Mean±SD		
		PWO+PWE (N)	PWO (N)	PWE (N)
Mild	-SRI	10.04±2.74 (31)	10.55±2.69 (14)	9.62±2.79 (17)
	+SRI	8.9±2.64 (17)	8.07±1.80 (6)	9.35±2.98 (11)
	Total	9.55±2.74 (48)	9.81±2.68 (20)	9.51±2.82 (28)

Moderate	-SRI	22.07±4.83 (18)	21.51±5.72 (11)	22.96±3.18 (7)
	+SRI	20.65±5.24 (18)	22.11±5.63 (9)	19.19±4.66 (9)
	Total	21.36±5.02 (36)	21.78±5.54 (20)	20.84±4.40 (16)

Severe	-SRI	54.57±18.75 (31)	56.61±16.55 (21)	50.29±23.10 (10)
	+SRI	68.62±16.40 (10)	71.73±18.34 (7)	61.37±9.40 (3)
	Total	58.00±19.02 (41)	60.39±17.95 (28)	52.85±20.94 (13)

AHI: apnea-hypopnea index, OSA: obstructive sleep apnea,

SRI: serotonin reuptake inhibitor, PWO: people without epilepsy,

PWE: people with epilepsy, and SD: standard deviation.

**Table 4:** Outcomes for total cohort: +SRI compared to -SRI.

Outcome		Covariate	p	OR	CI
Severe OSA compared to Moderate OSA	Unadjusted*∗*	------	0.022	0.323	0.123-0.848
	Univariate*∗*	Age	0.021	0.317	0.120-0.838
	Univariate*∗*	AED	0.027	0.333	0.126-0.884
	Multivariate*∗*	Age, AED	0.025	0.325	0.121-0.870

Severe OSA compared to Mild OSA	Unadjusted	------	0.262	0.588	0.233-1.485
	Univariate	Age	0.146	0.489	0.187-1.282
	Univariate	AED	0.556	1.339	0.507-3.537
	Multivariate	Age, AED	0.346	1.625	0.592-4.458

SRI: serotonin reuptake inhibitor, AED: antiepileptic drug, AHI: apnea-hypopnea index, PWO: people without epilepsy, PWE: people with epilepsy, and COPD: chronic obstructive pulmonary disease; *∗* = p<0.05.

Covariate: the listed characteristic(s) designates the variable(s) adjusted for in logistic regression analysis.

**Table:** (a) Outcomes for PWE versus PWO: +SRI compared to -SRI

Outcome		Covariate	p	OR	CI
Severe OSA compared to Moderate OSA	Unadjusted	------	0.079	0.233	0.046-1.185
	Univariate	Age	0.106	0.238	0.042-1.355
	Univariate	DM	0.083	0.469	0.026-5.395
	Univariate	COPD/asthma	0.052	0.127	0.016-1.018
	Univariate*∗*	AED	0.024	0.319	0.118-0.858
	Multivariate*∗*	Age, DM, COPD/asthma, AED	0.015	0.264	0.091-0.770

Severe OSA compared to Mild OSA	Unadjusted	------	0.314	0.464	0.104-2.071
	Univariate	Age	0.203	0.361	0.075-1.731
	Univariate	DM	0.272	0.417	0.087-1.990
	Univariate	COPD/asthma	0.266	0.412	0.087-1.964
	Univariate	AED	0.447	1.471	0.545-3.970
	Multivariate	Age, DM, COPD/asthma, AED	0.234	1.922	0.656-5.631

SRI: serotonin reuptake inhibitor, AED: antiepileptic drug, AHI: apnea-hypopnea index, PWO: people without epilepsy, PWE: people with epilepsy, COPD: chronic obstructive pulmonary disease, and DM: diabetes; *∗* = p<0.05. Covariate: the listed characteristic(s) designates the variable(s) adjusted for in logistic regression analysis.

**Table:** (b) Outcomes for PWO: +SRI compared to -SRI

Outcome		Covariate	p	OR	CI
Severe OSA compared to moderate OSA	Unadjusted	------	0.152	0.407	0.119-1.391
	Univariate	COPD/asthma	0.085	0.302	0.077-1.180

Severe OSA compared to mild OSA	Unadjusted	------	0.701	0.778	0.216-2.806
	Univariate	COPD/asthma	0.579	0.687	0.182-2.591

SRI: serotonin reuptake inhibitor, PWO: people without epilepsy, and COPD: chronic obstructive pulmonary disease.

**Table 6:** Outcomes for PWE: +SRI compared to -SRI.

Outcome		Covariate	p	OR	CI
Severe OSA compared to moderate OSA	Unadjusted	------	0.079	0.233	0.046-1.185
	Univariate*∗*	Age	0.042	0.140	0.021-1.116

Severe OSA compared to mild OSA	Unadjusted	------	0.314	0.464	0.104-2.071
	Univariate	Age	0.203	0.361	0.075-1.730

SRI: serotonin reuptake inhibitor and PWE: people with epilepsy; *∗* = p<0.05. Covariate: the listed characteristic designates the variable adjusted for in logistic regression analysis.

## Data Availability

The data used to support the findings of this study were provided by the site institution and so cannot be made freely available. Access to these data will be considered by the author upon request, with permission of the NYU School of Medicine Institutional Review Board.
